# Enhanced Separation of Palladium from Nuclear Wastewater by the Sulfur-Rich Functionalized Covalent Organic Framework

**DOI:** 10.3390/nano15100714

**Published:** 2025-05-09

**Authors:** Junli Wang, Chen Luo, Wentao Wang, Hui Wang, Yao Liu, Jianwei Li, Taihong Yan

**Affiliations:** 1 Department of Radiochemistry, China Institute of Atomic Energy, Beijing 102413, China; jlwang92@163.com (J.W.); wanghui1982sc@gmail.com (H.W.); 18801256495@163.com (Y.L.); 2 State Key Laboratory of Chemical Resource Engineering, College of Chemical Engineering, Beijing University of Chemical Technology, Beijing 100029, China; luochen00113@163.com

**Keywords:** COF, TAPB-BMTTPA-COF, adsorption, Pd (II)

## Abstract

The separation of palladium from radioactive waste streams represents a critical aspect of the secure handling and disposal of such hazardous materials. Palladium, in addition to its radioactive nature, holds intrinsic value as a resource. Despite the urgency, prevailing adsorbents fall short in their ability to effectively separate palladium under highly acidic environments. To surmount this challenge, our research has pioneered the development of 1,3,5-tris(4-aminophenyl)benzene-2,5-Bis(methylthio)terephthalaldehyde COF (TAPB-BMTTPA-COF), a novel material distinguished by its remarkable stability and an abundance of sulfur-containing functional groups. Leveraging the pronounced affinity of the soft ligands’ nitrogen and sulfur within its molecular architecture, TAPB-BMTTPA-COF demonstrates an exceptional capability for the selective adsorption of palladium. Empirical evidence underscores the material’s swift adsorption kinetics, with equilibrium achieved in as little as ten minutes, and its broad tolerance to varying acidity levels ranging from 0.1 to 3 M HNO_3_. Furthermore, TAPB-BMTTPA-COF boasts an impressive adsorption capacity, peaking at 343.6 mg/g, coupled with high selectivity in 13 interfering ions’ environment and the ability to be regenerated, making it a sustainable solution. Comprehensive analyses, including Fourier Transform Infrared Spectroscopy (FT-IR) and X-ray photoelectron spectroscopy (XPS), alongside Density Functional Theory (DFT) calculations, have corroborated the pivotal role played by densely packed nitrogen and sulfur active sites within the framework. These sites exhibit a robust affinity for Pd(II), which is the cornerstone of the material’s outstanding adsorption efficacy. The outcomes of this research underscore the immense potential of COFs endowed with resilient linkers and precisely engineered functional groups. Such COFs can adeptly capture metal ions with high selectivity, even in the face of severe environmental conditions, thereby paving the way for the more effective and environmentally responsible management of radioactive waste.

## 1. Introduction

Palladium (Pd), a member of the illustrious platinum group metals (PGMs), stands out for its remarkable physical and chemical attributes [1], serving as a cornerstone in diverse industries ranging from catalysis [2] and electronics manufacturing [3] to pharmaceuticals [4], corrosion-resistant materials [5], and exquisite jewelry [6]. Yet, the rarity of palladium and its scant presence in the Earth’s crust [7], compounded by the escalating environmental consciousness, most notably the burgeoning demand for palladium in automotive catalysts [8], have not only strained its supply but also underscored its strategic significance in the global market [9].

Dominated by a select few nations, such as South Africa and Russia, the world’s PGM reserves render other countries largely dependent on imports [10]. In response to this challenge, the extraction of palladium from secondary sources like electronic waste [11], spent catalysts [12], and nuclear byproducts [13] emerges as a critical solution. This approach not only reduces reliance on depletable resources but also boosts the circular economy through resource recycling. Notably, the retrieval of palladium from radioactive waste, particularly that generated by the reprocessing of spent nuclear fuel, which abounds in PGM ions, represents a fertile research advancement [14,15]. The recovery of palladium from high-level liquid waste (HLLW) not only augments palladium stocks but also streamlines subsequent vitrification processes, given palladium’s high melting point, which can otherwise complicate its integration with glass matrices [16,17,18].

In the realm of metal ion separation and recovery from radioactive waste, solvent extraction [19] and solid-phase adsorption techniques [20] dominate the literature. While solvent extraction, epitomized by the PUREX process for uranium and plutonium separation [21], is prevalent, adsorption methods outshine in scenarios involving low palladium concentrations due to their superior mass transfer efficiency, reduced secondary waste, and operational ease [22]. Researchers have engineered a plethora of solid-phase adsorbents, including silica-based materials [23] and porous organic polymers [24], and have strategically incorporated soft atoms such as nitrogen and sulfur to amplify their adsorption prowess and selectivity for palladium [25,26].

Covalent organic frameworks (COFs), with their organic porous crystalline architecture [27], have recently emerged as versatile materials, promising in separation, adsorption, transport, sensing, energy storage, and catalysis [28,29,30,31]. COFs boast high adsorption capacities, expansive surface areas, and customizable pores, coupled with exceptional acid resistance and stability, rendering them uniquely suited for heavy metal remediation [32,33].

Leveraging this background, this study introduces a sulfur-containing COF (TAPB-BMTTPA-COF) synthesized for palladium separation in nitric acid environments. The material demonstrates exceptional adsorption capabilities across a wide range of nitric acid concentrations (0.1 to 3 M), exhibits robust acid resistance, and conforms to the Langmuir adsorption model with a theoretical adsorption capacity of 343.6 mg/g. Moreover, it demonstrates high selectivity for 14 coexisting ions and excellent regenerability, positioning it as a premier candidate for the efficient extraction and recovery of palladium from radioactive waste.

## 2. Materials and Method

### 2.1. Materials

1,3,5-tris(4-aminophenyl)benzene (TAPB, purity exceeding 93%) was procured from Beijing InnoChem Science & Technology Co., Ltd., situated in Beijing, China. The laboratory solvents, including o-dichlorobenzene (o-DCB), 1-butanol (n-BuOH), acetone, chloroform (CHCl_3_), tetrahydrofuran (THF), and 1,4-dioxane, were all sourced from Shanghai Aladdin Biochemical Technology Co., Ltd., based in Shanghai, China. Potassium carbonate (K_2_CO_3_) and anhydrous hydrazine were acquired from Sinopharm Chemical Reagent Co., Ltd., also located in Shanghai, China. The remaining reagents utilized were of analytical grade and employed as received without additional purification.

### 2.2. Preparation of TAPB-BMTTPA-COF

The synthesis of 2,5-Bis(methylthio)terephthalaldehyde (BMTTPA) was executed via the solvothermal method, as previously documented in references [34,35]. The procedure unfolded as follows: Initially, a proportioned mixed solution was assembled, comprising 5 mL each of o-dichlorobenzene (o-DCB) and n-butanol (BuOH), complemented by a 1 mL addition of 6 M acetic acid (AcOH). This blend was then enriched with a precise measure of 35 mg of TAPB and 75 mg of BMTTPA, which were seamlessly integrated into the solution. The resultant mixture was then transferred into a 20 mL Pyrex test tube. The test tube, once sealed, underwent a controlled heating process at a consistent temperature of 120 °C for a duration of 72 h. Post-reaction, the precipitate was isolated through centrifugation and underwent a thorough cleansing process, alternating between washes with anhydrous tetrahydrofuran (THF) and acetone to effectively purge any residual impurities. The final step involved drying the purified precipitate overnight at 120 °C under vacuum conditions, culminating in the pristine acquisition of TAPB-BMTTPA-COF powder. This synthesis process ensured the purity and integrity of the final product, adhering to the rigorous standards of chemical synthesis.

### 2.3. The Adsorption Performance of TAPB-BMTTPA-COF Towards Pd(II)

To commence the procedure, a standard palladium solution is prepared, containing 10 g/L Pd(II), dissolved in 1 M HNO_3_. The initial solution’s acidity and palladium concentration are then fine-tuned using 6 M HNO_3_ or deionized water, ensuring precise proportions. Subsequently, an appropriate quantity of TAPB-BMTTPA-COF powder is introduced into a 15 mL centrifuge tube, which has been pre-filled with 10 mL of a solution specifically adjusted for acidity and Pd(II) concentration. The tube is then placed in a controlled environment at a constant temperature of 25 °C, where it undergoes a 12 h adsorption experiment, subjected to a consistent shaking speed of 280 rpm. Upon the completion of the experiment, 2 mL of the solution is extracted and passed through a 0.22 μm filter membrane to eliminate any particulate matter. The filtrate is then diluted with 0.1 M of HNO_3_ to reach a total volume of 10 mL, setting the stage for subsequent analysis. The diluted solution is subjected to a highly sensitive inductively coupled plasma optical emission spectrometry (ICP-OES) analysis. This advanced technique allows for the precise determination of the residual Pd(II) concentration within the solution, ensuring the accuracy and reliability of the experimental results.

The adsorption process was monitored and calculated by the equilibrium adsorption capacity (*q*_e_, mg/g) and partition coefficient (*K*_d_, mL/g) according to the following equations.

(1) $$q_{e}=\frac{(C_{i}-C_{e})\times V}{m}$$

(2) $$K_{d}=\frac{C_{i}-C_{e}}{C_{e}}\times \frac{V}{m}$$

where *V* is the volume of the solution (mL), *m* is the amount of adsorbent (g), and *C_i_* and *C_e_* are the initial and final equilibrium concentration of Pd(II), respectively.

### 2.4. Characterizations

The intricate structures, components, and stability of the materials were assessed through a series of sophisticated analytical techniques. Powder X-ray diffraction (PXRD) analysis was conducted using a SmartLab 3 KW instrument, employing Cu Kα radiation (λ = 1.5406 Å) with a scanning speed of 0.1°/min over the 2θ range of 3° to 30°. Scanning electron microscopy (SEM) with a Hitachi Regulus 8100 system, in conjunction with energy dispersive X-ray spectroscopy (EDX), provided detailed morphological and elemental characterizations. The adsorption properties were evaluated through N_2_ sorption isotherms, facilitated by a liquid N_2_ bath on a Micromeritics/3FLEX apparatus. Thermal stability was examined via thermogravimetric analysis (TGA/DSC3+, Mettler Toledo, Zurich, Switzerland) from ambient temperature to 800 °C at a heating rate of 10°/min under a protective N_2_ atmosphere. Infrared spectroscopic analysis was performed using a Nicolet 6700 FT-IR spectrometer (Thermo Fisher Scientific, Waltham, MA, USA). Elemental composition was further scrutinized by an Inductively Coupled Plasma Optical Emission Spectrometer (ICP-OES, JY2000-2, HORIBA, Paris, France). Lastly, the surface chemistry was elucidated by X-ray photoelectron spectroscopy utilizing a Thermo ESCALAB 250 spectrometer (Thermo Fisher Scientific, Waltham, MA, USA) equipped with Al Kα irradiation at θ = 90°.

### 2.5. Density Functional Theory Calculation

To delve deeper into the intricacies of bonding energy between Pd(II) and specific functional groups, a series of sophisticated DFT calculations were conducted. Employing the Vienna Ab Initio Simulation Package (VASP) as the computational tool, the complex interactions [36,37] were analyzed. The core ionic structures were precisely modeled using projected augmented wave (PAW) potentials, ensuring an accurate representation of the atomic cores [38,39]. To accommodate the electronic framework, a plane wave basis set was utilized, setting the kinetic energy cutoff at 450 eV for optimal resolution. To manage the partial occupancies of the Kohn–Sham orbitals, the Gaussian smearing method was implemented, which facilitated a more realistic simulation of the electronic distributions. To account for dispersion interactions, which are crucial in molecular systems, we integrated Grimme’s DFT-D3 methodology into our simulations [40,41]. This addition significantly enhanced the accuracy of our calculations by capturing subtle, long-range interatomic forces. Throughout the computational process, the Brillouin zone was efficiently sampled using a gamma-centered grid with dimensions of 1 × 1 × 1, ensuring comprehensive coverage of the electronic states [42]. The adsorption energy (*E*_ads_) was then calculated using a precise formula, which is pivotal for quantifying the strength of the interactions between Pd(II) and the special groups under investigation.

*E_ads_* = *E_total_* − *E_TAPB-BMTTPA-COF_* − *E*_ions_ (3)

where *E_total_*, *E_TAPB-BMTTPA-COF_*, and *E_ions_* are the energies of the ions system, the TAPB-BMTTPA-COF, and the isolated ions, respectively.

## 3. Results and Discussion

### 3.1. Characterizations of TAPB-BMTTPA-COF

**Figure 1:**
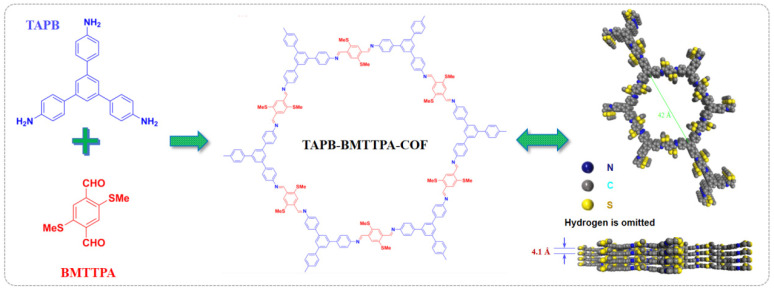
Synthetic scheme of TAPB-BMTTPA-COF and its graphic view.

This article presents a detailed analysis of the TAPB-BMTTPA-COF, a material characterized by its ordered mesoporous 1D channels and methyl sulfide units that serve as active sites for anchoring Pd(II). Figure 1 illustrates this unique structure, highlighting its potential applications.

**Figure 2:**
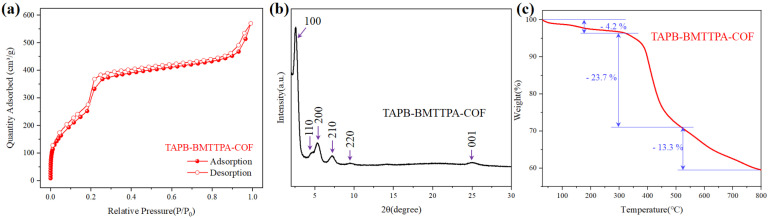
(**a**) Nitrogen sorption isotherm, (**b**) PXRD pattern, and (**c**) TGA curves of TAPB-BMTTPA-COF.

To delve deeper into the structural properties of the TAPB-BMTTPA-COF, various characterization techniques were employed, as depicted in Figure 2. Specifically, the nitrogen sorption isotherm at 77 K yielded a substantial Brunauer−Emmett−Teller (BET) surface area of 585 m^2^/g, indicative of Type IV behavior, as shown in Figure 2a. This result underscores the material’s high surface area, which is crucial for its functionality. The crystalline nature of the TAPB-BMTTPA-COF is further elucidated by the X-ray diffraction (XRD) pattern presented in Figure 2b. The pattern exhibits distinct peaks at angles 2θ = 2.72° (d = 32.5 Å), 4.81°, 5.53°, 7.38°, 9.72°, and 25.37°, corresponding to the (100), (110), (200), (210), (220), and (001) planes, respectively. These findings confirm the material’s high crystallinity, a property that is indispensable for its practical applications. The lattice spacing, *d*, was calculated using the Bragg equation [43]. Thermogravimetric analysis (TGA), as shown in Figure 2c, reveals the TAPB-BMTTPA-COF’s remarkable thermal stability, persisting up to 400 °C under nitrogen. Subsequent weight losses of approximately 24% between 400 °C and 520 °C are attributed to the decomposition of functional groups and the decomposition of the framework. Beyond 520 °C, a further 13% weight loss signifies the disintegration of the main framework.

Collectively, these comprehensive characterizations affirm that the TAPB-BMTTPA-COF exhibits a robust structure, a significant specific surface area, and the ability to maintain its crystalline integrity over a wide temperature range. These attributes position the TAPB-BMTTPA-COF as a promising material for a variety of applications.

### 3.2. TAPB-BMTTPA-COF Sorption Performance Towards Pd(II)

**Figure 3:**
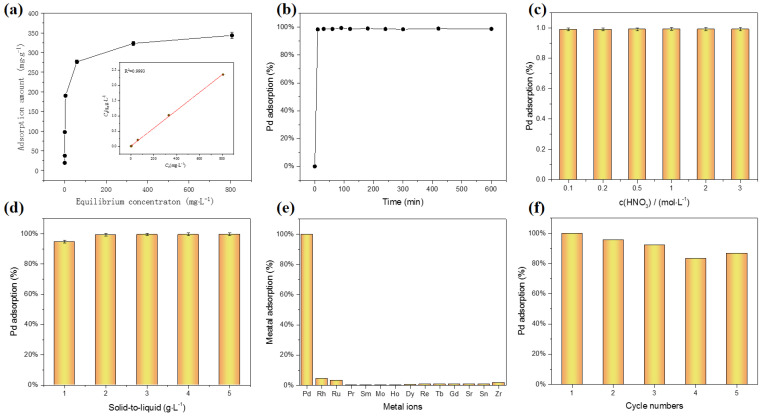
(**a**) The adsorption isotherm of TAPB-BMTTPA-COF towards Pd (II) with the fitted line by the Langmuir adsorption model in the inset. (**b**) Adsorption kinetic process of TAPB-BMTTPA-COF towards Pd(II). Effect of (**c**) HNO_3_ concentration on the adsorption of TAPB-BMTTPA-COF. (**d**) The correlation between TAPB-BMTTPA-COF dose and adsorption capacity. (**e**) The partition coefficient (*K*_d_) of TAPB-BMTTPA-COF towards various ions. (**f**) The TAPB-BMTTPA-COF reusability.

This study further examined the adsorption characteristics of the TAPB-BMTTPA-COF material with respect to Pd(II), as presented in Figure 3. Initially, the adsorption isotherm of TAPB-BMTTPA-COF was rigorously assessed to determine its capacity to adsorb Pd(II) in 3 M HNO_3_ solution medium. This evaluation was designed to measure the material’s adeptness in capturing Pd(II) ions under acidic conditions. As illustrated in Figure 3a, the analysis revealed a distinct correlation between the equilibrium concentration of Pd(II) and the adsorption capacity of the material. Importantly, the data points closely aligned with the classical Langmuir model (R^2^ = 0.9993), a fact depicted in the inset of Figure 3a. This alignment with the Langmuir model emphasizes the monolayer adsorption nature of TAPB-BMTTPA-COF on Pd(II), thereby underscoring its potential applicability in scenarios that demand selective and efficient Pd(II) capture under acidic conditions.

(4) $$\mathrm{Langmuir}\;\mathrm{model}:\frac{C_{e}}{q_{e}}=\frac{1}{q_{\max}K_{L}}+\frac{C_{e}}{q_{\max}}$$

In the formula, the parameters *q*_e_ and *q*_max_ (mg/g) delineate the adsorbent’s equilibrium adsorption capacity and its theoretical maximum adsorption threshold, respectively. *C*_e_ (mg/L) represents the concentration of metal ions in the aqueous phase at adsorption equilibrium. *K*_L_ (mg/L) emerges as a pivotal constant within the Langmuir adsorption model, shedding light on the intensity of the interaction between the adsorbent and the adsorbed substance. According to the formula, the theoretical maximum adsorption capacity is 343.6 mg/g.

In addition, this study also delved deeply into the adsorption process and its kinetic characteristics. As shown in Figure 3b, the adsorption mechanism of Pd(II) by the TAPB-BMTTPA-COF material exhibits an initial rapid phase, transitioning into a more gradual kinetic process, ultimately reaching over 99.5% of its equilibrium adsorption capacity within a mere 10 min. This swift uptake is attributed to the intricate pore structure and the presence of surface S-containing groups within the TAPB-BMTTPA-COF, which facilitate the rapid entrapment of Pd(II). As the adsorption process unfolds, the available sites for adsorption gradually become saturated, causing a deceleration in the rate of adsorption until equilibrium is attained.

In addition, the adsorption capabilities of TAPB-BMTTPA-COF were comprehensively examined across a spectrum of acidity levels by introducing varying concentrations of HNO_3_, ranging from 0.1 to 3 M, as depicted in Figure 3c. The findings reveal that this material exhibits exceptional adsorption efficiency consistently across a broad pH range. As previously noted, TGA confirmed its robust thermal stability, and now this study also highlights its pronounced chemical stability. This dual stability underscores the robust nature of the COF, characterized by its high crystallinity. Such attributes strongly suggest the substantial potential of TAPB-BMTTPA-COF for enduring and the effective adsorption of Pd(II) ions, even in the demanding conditions of highly acidic environments. This not only affirms its suitability for practical applications but also highlights its resilience and reliability in long-term usage scenarios.

The dosage of an adsorbent plays a pivotal role in determining its adsorption efficiency, thereby influencing its economic viability. This study delves into the intricate relationship between the adsorbent dosage and its performance in adsorption processes. As depicted in Figure 3d, a marked enhancement in the adsorption rate was observed when the dosage of TAPB-BMTTPA-COF was elevated from 1 g/L to 2 g/L. During this increment, the adsorption rate for palladium experienced a notable surge, escalating from 94.7% to an impressive 99.2%. However, beyond this threshold, further augmentations in the adsorbent dosage yielded minimal improvements, with the adsorption rate inching up to merely 99.5%. These findings underscore the efficacy of employing 2 g of adsorbent per liter of palladium-containing waste liquid to achieve the optimal adsorption of palladium, suggesting a cost-effective and efficient approach to managing such waste.

The practical utility of TAPB-BMTTPA-COF in capturing Pd(II) is significantly influenced by its performance across diverse metal ion environments. At room temperature, an evaluation under a 3 M HNO_3_ solution reveals the distinct adsorption rates of various metal ions, as depicted in Figure 3e. Notably, under identical conditions, TAPB-BMTTPA-COF exhibits an exceptional adsorption rate of 99.8% for Pd(II), underscoring its formidable capacity for the removal and separation of this metal. Conversely, the adsorption rates for other metal ions, including Rh, Ru, Pr, Sm, Mo, Ho, Dy, Re, Tb, Gd, Sr, Sn, and Zr, remain notably low, thereby highlighting TAPB-BMTTPA-COF’s pronounced selectivity for Pd(II). This remarkable selectivity can be attributed to the presence of the chelating group -S, which boasts a robust affinity for Pd(II), and the inherent structural features of TAPB-BMTTPA-COF. These include ample channels with flexible ligands and a framework with substantial porosity, which collectively enhance the material’s selectivity and adsorption capacity for Pd(II). This synergy not only elevates the material’s performance but also underscores its potential for practical applications in the effective separation and recovery of Pd(II), even in challenging environmental conditions. Thus, TAPB-BMTTPA-COF emerges as a promising candidate for enhancing the efficiency and selectivity of Pd(II) recovery processes.

**Figure 4:**
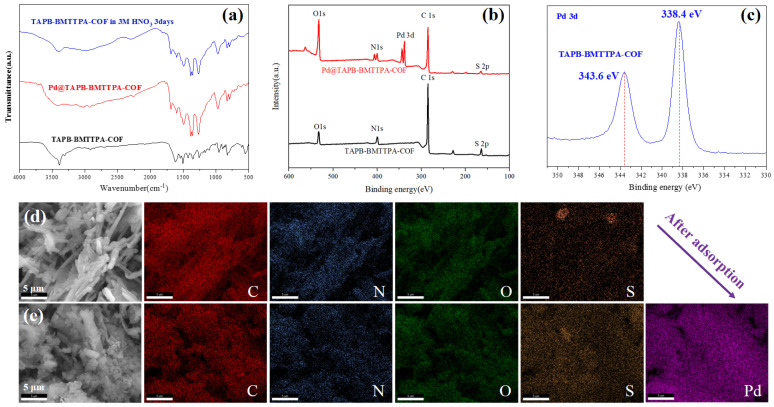
(**a**) FT-IR, (**b**) full XPS, and (**c**) Pd3d spectra of TAPB-BMTTPA-COF and Pd@TAPB-BMTTPA-COF. SEM images and elements distribution of (**d**) TAPB-BMTTPA-COF and (**e**) Pd@TAPB-BMTTPA-COF.

The economic significance of reusing the TAPB-BMTTPA-COF material cannot be overstated. To evaluate its reusability, this innovative material undergoes a treatment with thiourea, followed by incubation in a controlled environment, a shaking unit maintained at a constant 25 °C for a duration of 24 h. Post-incubation, the samples are subjected to centrifugation, thorough washing, and are subsequently deployed in a series of adsorption tests. The results, as depicted in Figure 3f, unequivocally demonstrate the exceptional recyclability of TAPB-BMTTPA-COF. Remarkably, even after undergoing five rigorous cycles, the material retains an impressive 86.9% of its original efficiency. This resilience is further underscored by the material’s steadfast stability under extreme conditions, specifically in the presence of a 3 M solution of HNO_3_. As depicted in Figure 4a, a 3-day immersion experiment of TAPB-BMTTPA-COF in 3 M acid is conducted, and the FITR characterizations of initial and final materials indicate that the structure of TAPB-BMTTPA-COF undergoes minimal changes after immersion, showcasing its durability in acidic environments.

The sulfur-rich TAPB-BMTTPA-COF material exhibits a remarkable potential for the selective adsorption of palladium, particularly due to its robust recyclability, which significantly enhances its utility across diverse applications. This exceptional performance can be ascribed to the material’s mesoporous channels and its high degree of crystallinity, features that are inherent to the TAPB-BMTTPA-COF structure. Moreover, the strategic incorporation of sulfur species within the TAPB-BMTTPA-COF is anticipated to foster significant binding interactions with Pd(II), thereby augmenting the material’s adsorption capabilities towards this metal. To elucidate the intricate mechanisms underlying this adsorption process, a suite of comprehensive characterizations, including Fourier Transform Infrared Spectroscopy (FT-IR), XPS, and SEM, are conducted and analyzed in the subsequent section. These analyses are poised to shed light on the nuanced interactions at play, further validating the material’s efficacy and potential in Pd adsorption applications.

### 3.3. The Sorption Mechanism of TAPB-BMTTPA-COF

The adsorption mechanism of TAPB-BMTTPA-COF and Pd@TAPB-BMTTPA-COF was further examined through FT-IR analysis. The FT-IR spectra, encompassing a range from 4000 to 500 cm^−1^ and depicted in Figure 4a, unveiled distinctive peaks indicative of the material’s molecular structure. Notably, a C=N stretching vibration peak at 1619 cm^−1^ was observed, attributable to the imine bond within the TAPB-BMTTPA-COF. Further insights were provided by the X-ray XPS spectra presented in Figure 4b. These spectra distinctly displayed peaks for O1s, N1s, and C1s, augmented by the emergence of a Pd3d peak post-adsorption, conclusively signaling the successful incorporation of Pd(II) into the material. The enhanced intensity of the Pd3d peak, as illustrated in Figure 4c, underscored the efficacy of Pd(II) adsorption.

**Figure 5:**
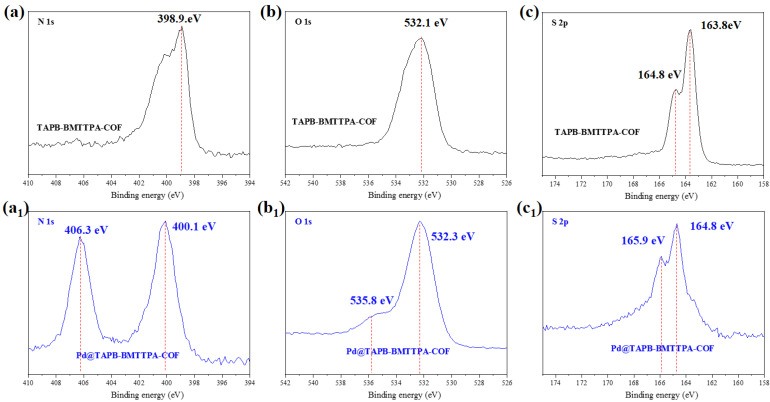
XPS spectra of N1s (**a**,**a1**), O1s (**b**,**b1**), and S2p (**c**,**c1**) for TAPB-BMTTPA-COF and Pd@TAPB-BMTTPA-COF.

Furthermore, the emergence of distinct new peaks in the N1 and O1 spectra, located at 406.3 eV and 535.8 eV, respectively, as illustrated in Figure 5a,b, unequivocally validates the formation of robust chemical bonds between Pd(II) and the TAPB-BMTTPA-COF. This deduction is further substantiated by the observed shifts in the S2p peaks, which transitioned from 163.8 eV to 164.8 eV and from 164.8 eV to 165.9 eV, as depicted in Figure 5c. These pivotal peak shifts are indicative of an enhanced chemical interaction between Pd and the TAPB-BMTTPA-COF, thereby bolstering the mechanism of chemical bonding. The SEM-EDS analysis provides insights into the morphological characteristics and elemental distribution within both the TAPB-BMTTPA-COF and Pd@TAPB-BMTTPA-COF, as showcased in Figure 4d,e. The fibrous samples exhibit remarkable stability subsequent to the adsorption of Pd(II), a fact inferred from the negligible morphological alterations observed during the adsorption process. Additionally, the uniform distribution of elements such as C, N, O, and S, as confirmed by EDS spectrometry, underscores the successful copolymerization and structural integrity maintained during adsorption. A noteworthy observation is the evenly distributed presence of Pd(II) elements following adsorption, which attests to the effective integration of Pd(II) within the TAPB-BMTTPA-COF structure.

**Figure 6:**
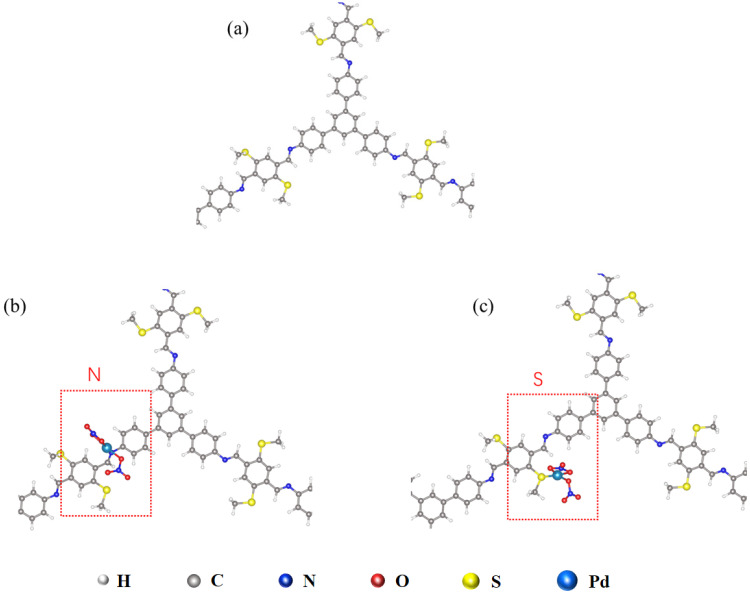
(**a**) TAPB-BMTTPA-COF structure and the DFT calculations of -Pd(NO_3_)_2_ with (**b**) N and (**c**) S on Pd@TAPB-BMTTPA-COF structure.

The efficient adsorption of Pd(II) by the TAPB-BMTTPA-COF material is primarily attributed to its tailored pore size and the strategic placement of functional groups within the COF, which facilitate robust interactions with Pd(II) ions. The diverse functional groups, such as nitrogen and sulfur, display varying binding affinities towards Pd(II) ions, reflecting differing binding energies. Interestingly, even identical functional groups, such as the C=N moiety, exhibit variable binding energies depending on the specific linker to which they are attached or their spatial orientation within the framework, thereby creating distinct chemical microenvironments [44]. Building upon the insights from Huang et al. [34], who posited that soft methylthio groups (-S-CH_3_) preferentially form bonds with soft heavy Hg(II) ions over imine bonds (N=C), this study corroborates the coordination interactions between Pd(II) and the methylthio units in TAPB-BMTTPA-COF through rigorous DFT analysis as shown in Figure 6. The adsorption of Pd(II) by TAPB-BMTTPA-COF samples was characterized by specific bond energies with nitrogen and sulfur, registering −1.83 eV for nitrogen and −2.00 eV for sulfur. This outcome underscores the formidable binding energy exhibited by the sulfur-rich TAPB-BMTTPA-COF structure with Pd(II). The chemical stability of this structure is bolstered by the presence of methyl sulfide units on the phenyl edges, which effectively mitigate the polarization-induced repulsions between the layers of the C=N bond [45]. In essence, this research positions the sulfur-rich TAPB-BMTTPA-COF structure as a highly promising candidate for the efficient separation and recycling of Pd(II), highlighting its potential in advanced materials for metal ion capture applications.

## 4. Conclusions

In this study, a highly stable and sulfur-rich covalent organic framework (COF), designated as TAPB-BMTTPA-COF, was synthesized and applied for palladium separation from radioactive wastes. Characterized by a 2D mesoporous architecture, this COF is distinguished by the strategic incorporation of –S-CH_3_ units along the phenyl edges, which significantly bolsters its chemical resilience. The framework’s abundant soft atoms of nitrogen and sulfur endow it a pronounced affinity for palladium. The experimental findings reveal that the TAPB-BMTTPA-COF demonstrates an impressive adsorption capacity for palladium within a 0.1–3 M HNO_3_ system, with its adsorption behavior aptly described by the Langmuir model. Remarkably, the material exhibits a robust adsorption capacity of 343.6 mg/g and manifests a high selectivity for palladium, with minimal adsorption of other competing ions. To delve deeper into the underlying interaction mechanism, this study employed XPS and DFT calculations. These analyses unequivocally highlight the pivotal role played by –S-CH_3_ in facilitating the formation of Pd-S coordination bonds via electron sharing. The collective results of this research underscore the promising potential of TAPB-BMTTPA-COF for the separation and recovery of palladium from radioactive wastes, thereby offering a significant advancement in the field of nuclear waste management.

## Data Availability

Data are contained within the article.
